# NetExtractor: Extracting a Cerebellar Tissue Gene Regulatory Network Using Differentially Expressed High Mutual Information Binary RNA Profiles

**DOI:** 10.1534/g3.120.401067

**Published:** 2020-07-14

**Authors:** Benafsh Husain, Allison R. Hickman, Yuqing Hang, Benjamin T. Shealy, Karan Sapra, F. Alex Feltus

**Affiliations:** ∗ Biomedical Data Science and Informatics Program, Clemson University, Clemson, SC; † Department of Genetics and Biochemistry, Clemson University, Clemson, SC; ‡ Center for Human Genetics, Clemson University, Clemson, SC; § Department of Electrical and Computer Engineering, Clemson University, Clemson, SC; ∗∗ Nvidia

**Keywords:** Mutual Information, Differential RNA, expression, Cerebellar gene, regulatory network

## Abstract

Bigenic expression relationships are conventionally defined based on metrics such as Pearson or Spearman correlation that cannot typically detect latent, non-linear dependencies or require the relationship to be monotonic. Further, the combination of intrinsic and extrinsic noise as well as embedded relationships between sample sub-populations reduces the probability of extracting biologically relevant edges during the construction of gene co-expression networks (GCNs). In this report, we address these problems via our NetExtractor algorithm. NetExtractor examines all pairwise gene expression profiles first with Gaussian mixture models (GMMs) to identify sample sub-populations followed by mutual information (MI) analysis that is capable of detecting non-linear differential bigenic expression relationships. We applied NetExtractor to brain tissue RNA profiles from the Genotype-Tissue Expression (GTEx) project to obtain a brain tissue specific gene expression relationship network centered on cerebellar and cerebellar hemisphere enriched edges. We leveraged the PsychENCODE pre-frontal cortex (PFC) gene regulatory network (GRN) to construct a cerebellar cortex (cerebellar) GRN associated with transcriptionally active regions in cerebellar tissue. Thus, we demonstrate the utility of our NetExtractor approach to detect biologically relevant and novel non-linear binary gene relationships.

Formulating gene co-expression networks (GCNs) or relevance networks Butte *et al.* (2000) is a powerful method to understand genetic relationships and explore biochemical mechanisms underlying phenotypic expression. A GCN consists of genes represented as nodes, where the relationships between genes, typically across a set of ribonucleic acid (RNA) expression profiles, are edges. A GCN is constructed from a gene expression matrix (GEM) where each edge is weighted by a value of interaction or correlation between the connected genes using a predefined metric. A threshold is then calculated to determine if the edge weight is significant to be included in the GCN. Eisen *et al.* (1998) developed the first reported GCN, and since then a variety of software tools have been implemented such as WGCNA Langfelder and Horvath (2008), CLR Faith *et al.* (2007), MRNET Peng *et al.* (2005), RMTGeneNet Gibson *et al.* (2013), KINC Ficklin *et al.* (2017), petal Petereit *et al.* (2016) and FastGCN Liang *et al.* (2015) using various approaches for identifying co-expression patterns.

Conventionally, GCNs leverage the Pearson or Spearman correlation metric to generate the weight of the edges in the network. As powerful as these techniques are to determine relationships between genes, they are severely restrictive since they primarily discover linear correlations and cannot detect non-linear or embedded relationships or require the RNA expression profiles to be monotonic. Also, it has been shown in previous works such as KINC Ficklin *et al.* (2017) and EdgeScaping Husain and Feltus (2019), that existing patterns can be masked by a combination of intrinsic and extrinsic noise, along with the possibility of multiple relationships existing for each gene-gene edge based on sub-populations of tissue samples. Hence, in recent years it can be observed that several new approaches at determining non-linear relationships within gene expression analysis has been explored. A common non-linear metric is mutual information (MI) Davies (1978) that has been utilized in a variety of applications by Roche *et al.* (2017), Barman and Kwon (2017), Pepke and Ver Steeg (2017), Zhang *et al.* (2014), Chan *et al.* (2017), and Wang *et al.* (2013).

In this report we aim to address the drawbacks of being limited to the detection of linear bigenic relationships for GCN construction, as well as the presence of noisy correlated edges, with NetExtractor. NetExtractor is a workflow that minimizes the problem of extrinsic noise with the application of Gaussian mixture models (GMMs), and explores non-linear latent relationships using the MI metric. MI essentially predicts the dependence of one random variable (RV) over another RV and this dependence is not restricted to linearity between gene expression relationships. It can also identify correlations in differential expressions in RNA-seq data within gene edges.

As a case study we apply NetExtractor to a GEM derived from brain tissue RNA expression profiles obtained from the Genotype-Tissue Expression (GTEx) project Lonsdale *et al.* (2013) where we extracted differential bigenic expression patterns in different brain tissue samples that were not detected with conventional correlation metrics. Applying MI over the GMMs permits us to explore novel non-linear relationships within sub-populations of samples. Leveraging these newly discovered gene-gene relationships as novel brain edges using NetExtractor along with the extensive brain gene regulatory networks (GRN) described by the PsychENCODE project Wang *et al.* (2018), we elucidate a GRN uniquely associated with cerebellar cortex (cerebellar) tissue gene expression.

## Materials And Methods

**Figure 1:**
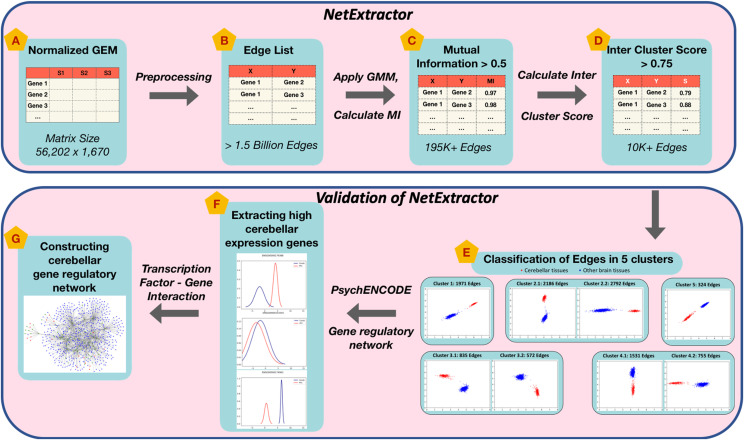
NetExtractor workflow and validation. Key stages of the algorithm are depicted by modules A-D. Modules E-G represent stages involved in the application and validation of NetExtractor using brain tissue RNA profiles from the Genotype-Tissue Expression (GTEx) project.

In this section we outline the NetExtractor workflow depicted in Figure 1, modules A-D. Source code for NetExtractor algorithm (Modules A-D) is available under the MIT license at https://github.com/bhusain/NetExtractor.git. Figure 1 modules E-G represent NetExtractor validation which is discussed in detail within the Result section.

### Gene Expression Matrix (GEM) normalization

Before applying the NetExtractor workflow to an RNAseq GEM, it is imperative to normalize the GEM. A parallel code was implemented on the Palmetto supercomputer at Clemson University for applying the following transformations:

Replace missing expression values for samples with ‘NA’.
Apply element-wise log2 transformation.
Identify and remove outlier samples using the K-S test (D < 0.15). Lopes *et al.* (2007)
Apply quantile normalization to ensure suitable comparison between samples. Rapaport *et al.* (2013)

**Figure 2:**
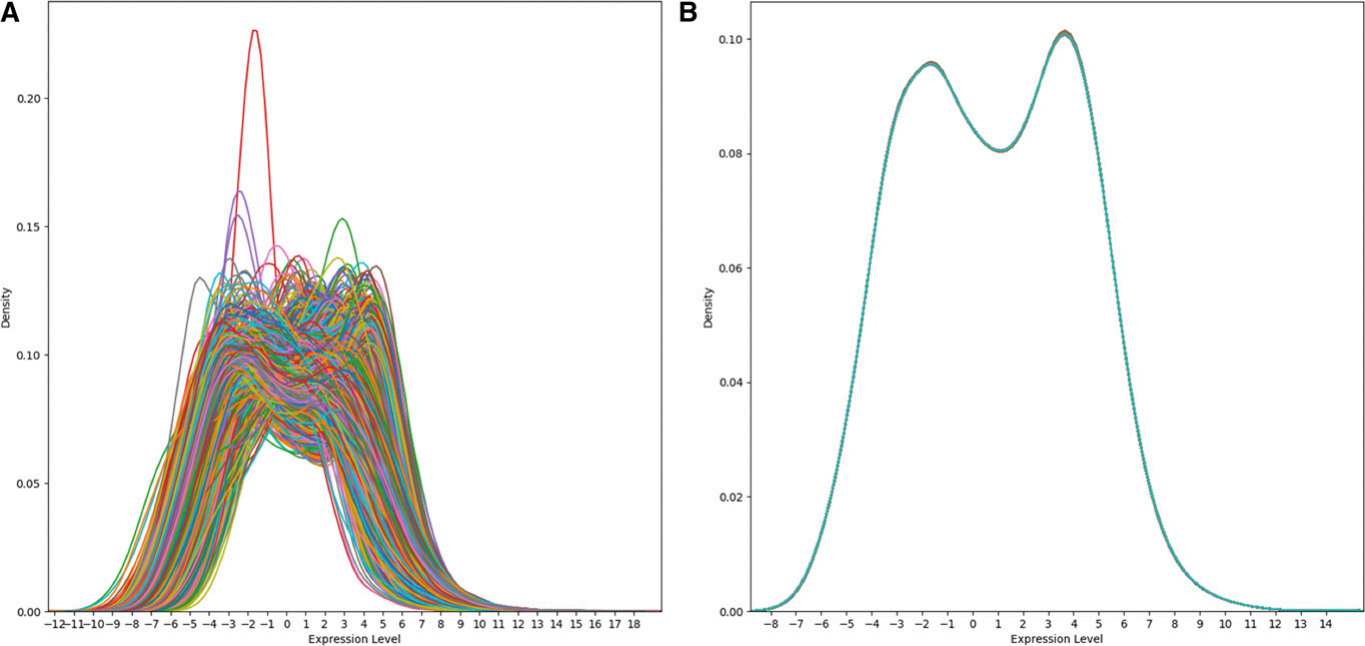
Normalization of panGTEx gene expression matrix. Sample distributions for panGTEx gene expression matrix before and after normalization.

We began with the panGTEx GEM Lonsdale *et al.* (2013) comprising of 56,202 genes along with 53 types of tissues being represented with 11,688 samples depicted by Figure 2. A similar normalization process was applied to the panTCGA GEM Hoadley *et al.* (2018) which contained 60,101 gene names along with 33 types of cancer being represented with 11,093 tissue samples. The K-S test eliminated a total of 328 outliers for panGTEx and 102 outliers in panTCGA GEMs.

Using the normalized panGTEx GEM, brain specific tissue samples were extracted into a brainGTEX GEM that consisted of 56,202 genes and 1,670 samples representing 13 brain tissue types [*Amygdala*, *Anterior cingulate cortex(BA24)*, *Caudate(basal ganglia)*, *Cerebellar Hemisphere*, *Cerebellum*, *Cortex*, *Frontal Cortex(BA9)*, *Hippocampus*, *Hypothalamus*, *Nucleus accumbens(basal ganglia)*, *Putamen(basal ganglia)*, *Substantia nigra*, *Spinal cord(cervical c-1)*] as depicted by Module A in Figure 1. Hence, the total number of possible edges for 56,202 genes to calculate the correlation weight is 1,579,304,301 edges as represented in Module B.

### NetExtractor workflow

In previous work, the KINC algorithm Ficklin *et al.* (2017) demonstrated the utility of using GMMs to sort expression states and address the problem of natural extrinsic (condition-specific) variation during GCN construction from mixed input expression conditions. The study illustrated that in gene-gene pairs with more than one condition-specific binary expression mode, the application of the Pearson or Spearman correlation metrics on the collective set of samples can be inappropriate to determine the true correlation value for a potential edge. Although utilization of GMMs in KINC shows promising results, a major drawback still persists where even within each mode only linear or monotonic relationships can be identified. In this study, we embrace the presence of multiple expression modes in a pairwise gene comparison as representative of condition-specific, possibly non-linear binary gene expression relationships. As with KINC, we identify multiple expression modes using GMMs. However, we replace correlation metrics with MI to detect non-linear relationships between gene pairs. Before we elaborate on the specifics on the implementation we give a brief overview of the background of the metrics utilized by NetExtractor.

***Gaussian Mixture Models (GMMs)*** GMMs is a probabilistic model that assumes all the data points are generated from a mixture of a finite number of Gaussian distributions with unknown parameters that incorporates information about the covariance structure of the data as well as the centers of the latent Gaussians. We utilized the python Scikit-learn ’sklearn.mixture.BayesianGaussianMixture’ Buitinck *et al.* (2013) implementation that differentiates into multiple classes to estimate GMMs that correspond to different estimation strategies. The Scikit-learn package implements the expectation-maximization (EM) algorithm for fitting mixture-of-Gaussian models. In the NetExtractor algorithm workflow, we applied GMMs to 1,579,304,301 possible brainGTEX edges to obtain clusters between 1-5 modes per edge using the variational Bayesian estimation of a Gaussian mixture. In our algorithm in order to investigate differential expression values we restricted our results to edges that contained 2 or more sub-population modes.

***Mutual Information (MI)*** MI is a measure of the similarity between two RVs, where $\left|U_{i}\right|$ is the number of the samples in cluster $U_{j}$ and $\left|V_{j}\right|$ is the number of the samples in cluster $V_{j}$. The MI between clusterings *U* and *V* is given as:

$$MI(U,V)=\sum_{i=1}^{\left|U\right|}\sum_{j=1}^{\left|V\right|}\frac{\left|U_{i}\cap V_{j}\right|}{N}log\frac{N\left|U_{i}\cap V_{j}\right|}{\left|U_{i}\right|\left|V_{i}\right|}$$

We utilize the the python Scikit-learn package to calculate the normalized MI score to scale the results between 0 (no mutual information) and 1 (perfect correlation). In NetExtractor, MI was calculated for all the gene pairs as well as for each individual sub-population modes per edge. The final value of MI assigned per edge is the average of the MI values for all individual GMM sub-population modes.

***Mean Silhouette Coefficient (S)*** Mean Silhouette Coefficient Rousseeuw (1987) is calculated using the mean intra-cluster distance *a* and the mean nearest-cluster distance *b* for each sample. The Silhouette Coefficient for a sample is then calculated as (b−a)/max(a,b). Essentially, *b* is the distance between a sample and the nearest cluster that the sample is not a part of. We utilize the ’sklearn.metrics.silhouettescore’ to calculate inter-cluster score between the different modes per edge. The best value is 1 and the worst value is -1. Values near 0 indicate overlapping clusters. Negative values generally indicate that a sample has been assigned to the wrong cluster, as a different cluster is more similar.

### MI based inter-cluster score thresholding

**Figure 3:**
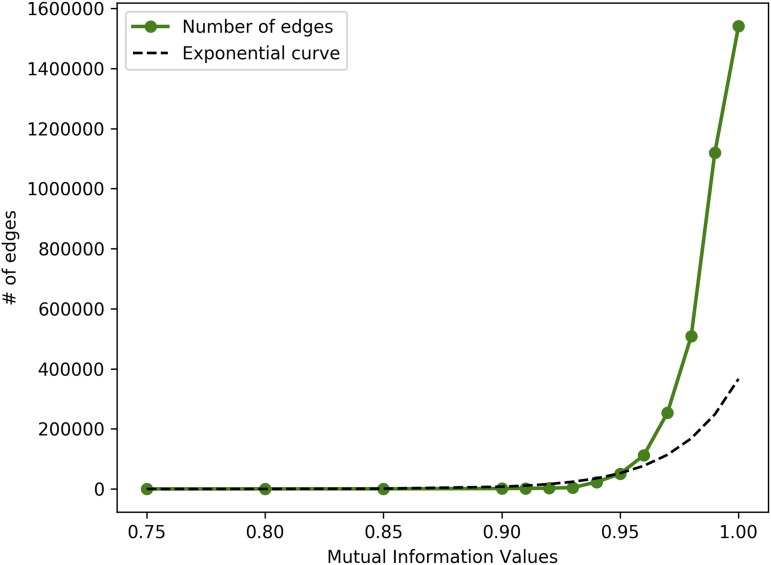
Mutual Information value distribution The distribution of MI values against the number of edges. We select a threshold MI >=0.95 based on the elbow of the distribution.

As depicted in Figure 1, we apply GMMs on the edge list which is then followed by calculating the MI for each sub-population mode and subsequently we calculate the inter-cluster score (S) for each gene-gene edge. As with other conventional methods we select two thresholds to restrict the edge that gets included in our GCN. The first threshold to be selected is the MI value that would entail the cutoff for the edge to be included within the GCN. For this purpose we randomly selected 5 million edges (experiment performed X 10 times) and plot the average of MI value distribution as depicted in Figure 3. We then select a value of threshold MI >=0.95 based on the observation that an exponential rise in MI can be noticed. It is essential to note since MI is a non-linear relationship metric that relies on the predictability of one RV based on the information of the second RV, it is to be expected for a large number of edges to contain high MI values. Basing NetExtractor solely on MI would result in a fairly large GCN but since we restrict edges that contain only two or more GMM sub-populations we further prune the network to 195,850 edges as shown by Module C in Figure 1. In order to restrict edges with differential expressions we ensured the GMM clusters are mutually exclusive and do not overlap in a significant manner. To this end we calculated the inter-cluster loss using the Mean Silhouette Coefficient (S) on the 195,850 edges and restrict the score to a threshold >= 0.75. This threshold was selected specifically to ensure that the overlap of the number of samples between the GMM sub-populations did not exceed twice the variance per sub-population. This resulted in a final edge list of 10,966 edges comprising 5,441 genes as depicted by Module D in Figure 1.

### Data availability

The normalized panGTEx and panTCGA GEMs along with their annotations data are hosted on the Clemson servers and are available upon request. Source code is available under the MIT license at https://github.com/bhusain/NetExtractor.git. S1 Table: Differentially expressed GCNs separated based on the five clusters. S2 Table: Functional enrichment analysis of all five cerebellar clusters. S3 Table: Subset of cerebellar associated GRN included 636 nodes connected via 1,146 edges. S4 Table: Functional enrichment output for WGCNA_GCN and KINC_GCN. Supplemental material available at figshare: https://doi.org/10.25387/g3.12649700.

## Results

When the 10,966 edges were visualized as scatter plots it was observed that all the edges had distinctly different expression levels for cerebellum and cerebellar hemisphere tissue samples when compared to other brain tissue samples. Thus, we further classified all these edges into 5 clusters based on how differential gene expression values were distributed. In the subsequent section we will elaborate on module E-G from Figure 1 as the application and validation of NetExtractor algorithm.

### Classification of cerebellar clusters

**Figure 4:**
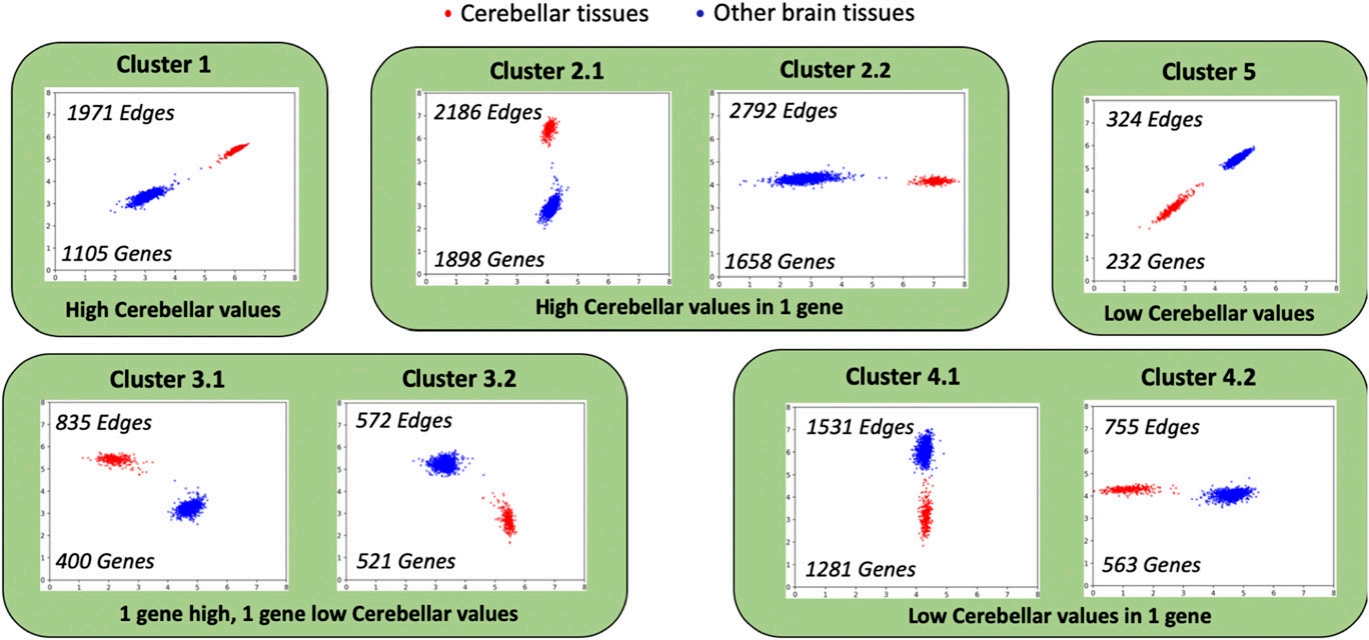
Classification of edges into clusters. Classification of edges into 5 clusters based on the distribution of cerebellar cortex tissues *vs.* non-cerebellar brain tissues differential expression values.

Based on the distribution of the expression levels between cerebellar *vs.* non-cerebellar samples, we manually categorized the edges into 5 broadly classified clusters as depicted by Figure 4 and included in Supplemental Table S1:

Cluster 1: Both genes had high cerebellar expression while low non-cerebellar expression levels. 1,971 edges and 1,105 genes were classified into this cluster.
Cluster 2: One of the genes in the edge had higher expression levels for cerebellar samples while the other gene has constant expression levels across all samples. 4,978 edges and 3,556 genes were classified into this cluster.
Cluster 3: One of the genes had high cerebellar expression levels and low non-cerebellar expression levels, while the other gene in the edge had the exact opposite expression level distributions. 1,407 edges and 921 genes were classified into this cluster.
Cluster 4: One of the genes in the edge had higher expression levels for non-cerebellar samples while the other gene had constant expression levels across all samples. 2,266 edges and 1,844 genes were classified into this cluster.
Cluster 5: Both genes had high non-cerebellar expression values and low cerebellar expression levels. Only 324 edges and 232 genes were classified into this cluster.

The overlap of the number of genes within each cluster can also be observed in Table 1. The total unique genes within all clusters is 5,441. It is noteworthy to observe that more edges containing higher cerebellar tissue expression levels were extracted using NetExtractor.

**Table 1:** Overlap of genes between the 5 clusters

	Cluster 1	Cluster 2	Cluster 3	Cluster 4	Cluster 5
Cluster 1	1105	265	382	206	0
Cluster 2		3556	316	988	99
Cluster 3			921	157	137
Cluster 4				1844	41
Cluster 5					232

### Functional enrichment analysis of cerebellar clusters

In order to investigate the cerebellar genes and edges picked using NetExtractor, we performed functional enrichment analysis based on functional annotations using the ToppGene suite Chen *et al.* (2009). For each cluster we counted the number of statistically significant enriched GO terms (q <10^−4^) for the GO categories: molecular function (MF), biological process (BP), and cellular component (CC). ToppGene human phenotype and microRNA labels were also analyzed.

It can be observed that clusters 1, 2, and 4 represent statistically significant functional enrichment as depicted by Table 2. On the other hand clusters 3 and 5 did not show significance in the enriched terms, and it is interesting to note that in both of these clusters both genes have differentially expressed values for cerbellar *vs.* non-cerebellar tissues with atleast one gene having a low expression profile for cerebellar tissue samples. It is also notable in Supplemental Table S2, which contains all of the enrichment information per cluster, that primary enriched terms for human phenotype for clusters 1, 2, and 4 were associated with human central nervous system phenotypes whereas clusters 3 and 5 were not enriched. We also note that nearly all the terms associated to the 5,441 genes that were analyzed were ’brain specific’, in fact displaying primary or secondary cerebellar functional activities. This phenomena could also be attributed to the fact that cluster 3 and 5 both contained relatively fewer genes than other clusters, which may have reduced the possibility of functional enrichment. Further investigation may be required to better understand why the differential distribution of cerebellar samples in both those clusters did not indicate strong ’brain specific’ terms. In order to validate the function enrichment results we perform two comparative analysis: (1) 100 sets of 5,000 random genes each extracted from the entire brainGTEX GEM to test statistical significance that did not lead to any enriched terms. (2) We take each cluster individually and perform functional enrichment while comparing against a similar number of random genes (random genes experiment performed X100).

**Table 2:** Functional enrichment analysis of the 5 categorized clusters

Gene Set	GO-MF	GO-CC	GO-BP	Human Phenotype	MicroRNA
Cluster1	12	17	69	44	3519
Cluster2	27	63	189	37	4914
Cluster3	1	7	7	0	3140
Cluster4	22	48	171	39	3933
Cluster5	0	0	0	0	388

### Cerebellar gene regulatory network construction

A pre-frontal cortex (PFC) GRN which describes significant transcription factor (TF) - target gene activity linkages was obtained from the PsycheENCODE project Wang *et al.* (2018). We utilized this PFC GRN to analyze the set of 5,441 genes that were obtained through the NetExtractor algorithm. There are two components to our decision to utilize the PsychENCODE PFC GRNs to create a cerebellar GRN. (1) The NetExtractor algorithm itself is knowledge independent, and based on the criteria of multiple mixture models with variance that are clearly non-overlapping, we discovered that within the brainGTEX GEM, cerebellar samples demonstrated the most distinct differential expression values. Hence, the decision to explore cerebellar associated gene pairs and construct a GRN was due to the output of NetExtractor. (2) We utilize the PsychENCODE datasets since it is one of the larger databases that compiles information about a significant number of genes with their transcription factors. One important caveat is that the database is specifically created for PFC tissues and not for cerebellar tissues. Since the genes we isolated through our technique have been shown to be specifically associated to cerebellar tissue, but the GRN information we have is for PFC, we used this opportunity to analyze the GRN in a unique context for genes that regulate cerebellar activity.

**Figure 5:**
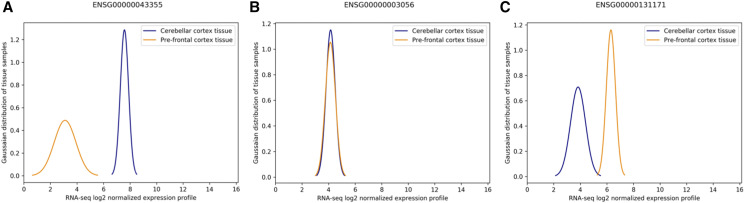
Tissue RNA profiles expressed as a Gaussian mixture model for cerebellar cortex (cerebellar) tissue *vs.* pre-frontal cortex (PFC) tissue. (A) An example distribution between the tissues where expression of cerebellar samples are significantly higher than PFC samples. (B) Example distribution where there is no significant difference between PFC and cerebellar tissue expression levels. (C) Example distribution between the tissues where expression of PFC samples are significantly higher than cerebellar samples.

The cerebellar gene set was pruned to only consider genes with regulatory information contained in the PsychENCODE PFC GRN. This reduced our gene list to 3,978. Since we were interested in genes that are specifically associated solely with cerebellar activity, we analyzed each of the 3,978 genes by isolating the PFC and cerebellar samples and performing an ANOVA test to determine whether the variance of expression levels between the samples of PFC *vs.* cerebellar tissue was significant. Figure 5A depicts genes with significantly higher expression levels for cerebellar tissues as opposed to PFC. In contrast Figure 5C has significantly lower expression values for cerebellar tissues than PFC, where as Figure 5B depicts no significant difference between the two expression level distributions. By eliminating all genes that did not represent a significant difference in expression levels to PFC, we further reduced the number of genes to 808. This step represents module F in Figure 1.

**Figure 6:**
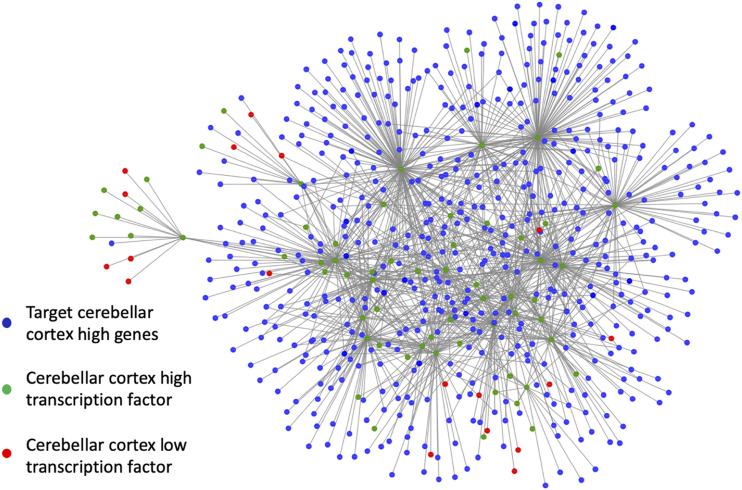
Cerebellar sub-gene regulatory network (GRN). The GRN constructed centered around genes that are both TFs as well as target genes with high cerebellar cortex (cerebellar) expression levels. Green nodes represent transcription factors (TF) with high cerebellar expression levels, red nodes represent TF with high pre-frontal cortex (PFC) expression levels, and blue nodes represent target cerebellar high genes.

In order to focus on a more manageable set of genes for the construction of a cerebellar GRN, we further pruned the list to primarily focus on genes that are both target genes (with higher cerebellar expression levels than PFC) as well as TFs represented by the green nodes in Figure 6. We then extracted the network of genes from the PFC PsychENCODE GRN that act as target genes to the green nodes. These target genes are represented by blue nodes in Figure 6. Similarly, any gene that is a TF to the green nodes as the target gene is also included in the network. Nodes colored red in Figure 6 indicate TF targeting the green nodes where the expression level for PFC is higher than that of cerebellar samples. In total, this subset of cerebellar associated GRN included 636 nodes connected via 1,146 edges. Details of this cerebellar GRN are provided in Supplemental Table S3.

### Cerebellar gene regulatory network validation

To validate the constructed cerebellar GRN, we compared the genes with a size-controlled random list for enrichment in transcriptionally active PsychENCODE histone acetylation peaks across three brain tissues. Since the PsychENCODE histone acetylation peaks were only annotated on autosomes and sex chromosomes, 7 out of 636 genes were dropped from the analysis due to their location on chromosome scaffolds. The remaining 629 are considered ’protein-coding’ genes by Ensembl Zerbino *et al.* (2017). A random list of genes was assembled using the *Homo sapiens* GRCh38 v85 GFF3 file from Ensembl as the gene pool. To be considered a random size-controlled match, a gene had to be ’protein-coding’, found on an autosome or sex chromosome, and have a gene length within 10% of an original gene. Genes in the original list were removed from the gene pool in order to avoid duplicates across lists.

We acquired the PsychENCODE coordinates for active histone acetylation peaks in cerebellar, PFC, and Temporal Cortex (TC) tissues. To encompass histone acetylation regions that may play a role in gene expression from an upstream or downstream position, 1000bp bumpers were added to both sides of each gene. With their respective extended coordinates, all original and size-controlled genes were then surveyed for overlaps with PsychENCODE histone acetylation peaks across all three brain tissues. Since in several cases it was observed that multiple histone acetylation peaks were active in more than one tissue, a tissue exclusive list was compiled that contained histone acetylation peaks exclusively active in only one of the three brain tissue types. Therefore, each gene in the original and size-controlled list was surveyed for overlap of histone acetylation peaks in the complete and exclusive lists for each tissue. We repeated randomized size-controlled list creation 100 times and determined the mean and variance to test for statistical significance between original and size-controlled genes. As a sanity check we also performed the same test by replacing the 629 genes from the cerebellar GRN by equal number of genes from the PsychENCODE PFC GRN, ensuring that these genes had high PFC expression levels in comparison to cerebellar tissue sample as depicted in Figure 5C.

**Figure 7:**
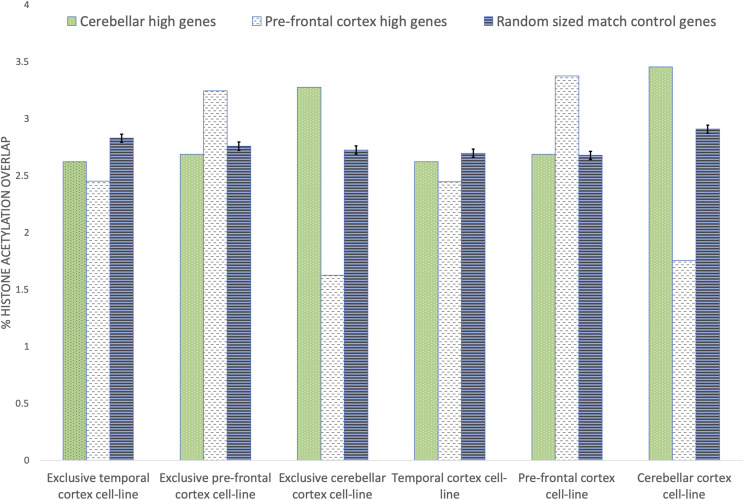
Brain tissue edge overlap with PsychENCODE histone acetylation peaks across three brain tissues. Comparing transcriptionally active PsychENCODE histone acetylation peaks between the extracted 629 cerebellar cortex (cerebellar) genes from the cerebellar sub-gene regulatory network, 629 pre-frontal cortex (PFC) genes that demonstrated higher expression values that cerebellar genes from the PsychENCODE GRN, and 629 random, length-control genes (n = 100). Exclusive refers to histone acetylation peaks active in only one of the three brain tissue types

The results for the active histone acetylation peaks overlap test are displayed in Figure 7 where the green bar represents the 629 cerebellar GRN genes, white bar represents the 629 PFC GRN genes, and blue bar indicates mean of 629 random size-controlled genes. It can be observed that the cerebellar GRN genes (green bar) culled using NetExtractor had significantly higher histone acetylation peak overlap for cerebellar and exclusive cerebellar tissues. Similarly, the PFC GRN genes (white bar) obtained from the PsychENCODE GRN have significantly higher histone acetylation peaks for PFC and exclusive PFC. By contrast when TC and exclusive TC tissues were tested, there was more overlap with active histone acetylation peaks in random size-controlled genes relative to cerebellar GRN or PFC GRN genes.

**Figure 8:**
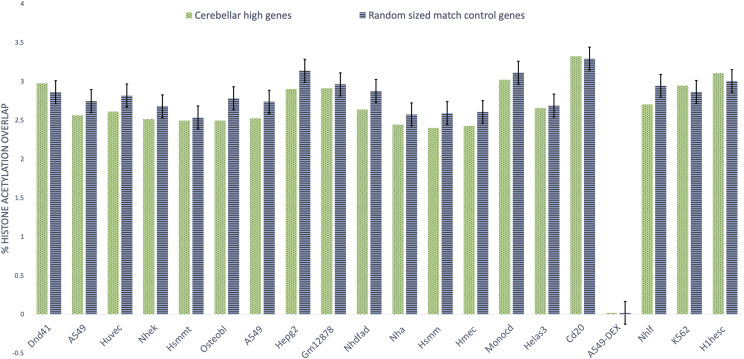
Brain tissue edge overlap with ENCODE histone acetylation peaks across 20 non-brain cell lines Comparing histone modifications across 20 *non-brain cell lines* between 629 cerebellar cortex (cerebellar) genes from the cerebellar sub-gene regulatory network and 629 random, length-control genes (n = 100).

To further validate the cerebellar GRN we compared the 629 genes for enrichment to a size-controlled 629 random gene list in 20 *non-brain cell lines* for histone modifications by ChIP-seq from the ENCODE project Bernstein *et al.* (2005) as depicted by Figure 8. The experiment was performed using the cerebellar GRN and compared against an equal number of randomized size-controlled genes (n = 100). In contrast to the earlier experiment it can be observed that in most cases the mean of random set of genes displayed similar histone acetylation peak overlap to the genes contained within cerebellar GRN. In summary, for validation of cerebellar GRN genes we utilize the histone acetylation peaks data in three ways: (1) Comparing genes within the cerebellar GRN against a similar number to random genes over 20 non-brain cell lines as represented by Figure 8. This validation experiment is aimed to demonstrate that in non-brain cell lines, random genes outperform for histone acetylation peaks than genes that we attribute to cerebellar GRN.(2) Comparing genes within the cerebellar GRN against a similar number to random genes as well as genes within the PFC GRN over three cell lines: cerebellar cortex, pre-frontal cortex, and temporal cortex. This experiment indicates that cerebellar GRN genes outperform PFC and random genes for histone acetylation peaks correspondence over cerebellar cortex genes. (3) Similarly, PFC genes have a significant overlap with histone acetylation peaks over pre-frontal cortex genes when compared against cerebellar and random genes. These set of experiments were performed as a validation test to check if the genes selected for the cerebellar GRN could infact be attributed to cerebellar cortex tissue. The interactions of edges within the GRN are validated based on the high MI values for each edge and the data essentially being a sub-network of the PsychENCODE PFC GRN.

### Comparing NetExtractor

In this manuscript we have illustrated the capability of utilizing NetExtractor to generate a cerebellar specific GCN as well as a GRN, but in order to elucidate the utility of differential gene expression using a non-linear relationships metrics it is essential to compare the output of NetExtractor to more conventional GCN construction techniques. For this purpose we utilize the WGCNA and KINC algorithms to generate WGCNA_GCN and KINC_GCN. In Table 4 we summarize the results comparing the outputs of the three algorithms. WGCNA essentially applies a weighted linear similarity measure to attribute a value for each gene-gene edge relationship. Hence, this correlation matrix is populated for the entire edgelist, while a threshold is selected to construct the final GCN. WGCNA pools all samples within an edge together to calculate the edge correlation score and is therefore a traditional linear metric for GCN construction. Along with the limitations of using a linear metric that are included in the manuscript, another drawback that was encountered is the large computation time it might require. We utilized IterativeWGCNA python wrapper implementation Greenfest-Allen *et al.* (2017) to construct WGCNA_GCN with the normalized GEM (Module A in Figure 1) as the input. Since,the significance threshold needed to be manually selected the default value of 0.5 was chosen to capture as many edges as possible with the result of 465,444 edges across 6,778 genes comprised within 85 modules as depicted by Table 4.

KINC on the other hand expands on the concept of WGCNA by first implementing GMMs to determine the existence of sub-populations within the samples. These sub-populations of selected edges can then be enriched in a tissue specific manner to attribute an edge to tissue types. Therefore, KINC does not club all the samples together while calculating relationships between the edges for gene-pairs.

**Table 3:** Top five enriched functions for genes in the cerebellar gene regulatory network (GRN)

Category	Term ID	Term Description	q-value
GO-MF	GO:0043565	sequence-specific DNA binding	2.36E-28
	GO:0003682	chromatin binding	5.19E-25
	GO:0001067	regulatory region nucleic acid binding	1.73E-21
	GO:0044212	transcription regulatory region DNA binding	3.10E-21
	GO:0008134	transcription factor binding	1.86E-20
GO-CC	GO:0044451	nucleoplasm part	1.10E-28
	GO:0005694	chromosome	3.66E-15
	GO:0044427	chromosomal part	3.67E-15
	GO:0000785	chromatin	6.26E-13
	GO:0016604	nuclear body	9.52E-12
GO-BP	GO:0010628	positive regulation of gene expression	4.58E-33
	GO:0006357	regulation of transcription by RNA polymerase II	1.35E-32
	GO:0051173	positive regulation of nitrogen compound metabolic process	1.37E-31
	GO:0045935	positive regulation of nucleobase-containing compound metabolic process	1.37E-31
	GO:0010557	positive regulation of macromolecule biosynthetic process	1.37E-31
Human Phenotype	HP:0008050	Abnormality of the palpebral fissures	2.26E-08
	HP:0200006	Slanting of the palpebral fissure	2.78E-07
	HP:0000315	Abnormality of the orbital region	5.28E-07
	HP:0000284	Abnormality of the ocular region	5.28E-07
	HP:0000527	Long eyelashes	6.98E-07
Diseases	C3714756	Intellectual Disability	5.53E-07
	C0038379	Strabismus	1.62E-05
	C0557874	Global developmental delay	8.46E-05
	C1864897	Cognitive delay	8.46E-05
	C4020875	Mental and motor retardation	8.46E-05

**Table 4:** Overlapping genes and edges between NetExtractor and other GCN construction methods

	KINC		KINC_Cerebellar		WGCNA	
	Genes	Edges	Genes	Edges	Genes	Edges
KINC	1,692	7,795	561	791	1,350	3,820
WGCNA			507	315	6,778	465,444
NetExtractor	375	0	122	0	2,774	295

KINC calculated the correlation for each gene pair upon using GMMs in a manner similar to NetExtractor. Only GMM sub-populations with equal to or more than 30 samples underwent Spearman correlation, while values less than 0 were ignored. 50,000 KINC similarity jobs were submitted on the Open Science Grid Roy *et al.* (2007) by using the OSG-KINC similarity workflow Poehlman *et al.* (2017). The KINC significance threshold of 0.8961 was found by using random matrix thresholding (RMT) algorithm within the KINC thresholding script. The KINC_GCN was then constructed by extracting the 7,795 edges comprised of 1,692 genes with correlations > 0.8961 contained within 183 linked community modules (LCM). All identified edges in the KINC_GCN were tested for sample label enrichment using the KINC.R package [https://github.com/SystemsGenetics/KINC.R], hence allowing us to calculate cerebellar specific edges (KINC_Cerebellar) as depicted in Table 4.

Finally, when discussing NetExtractor, theoretically it is a further extension of KINC where we not only apply GMMs but instead of using conventional linear or monotonic similarity metrics we use a non-linear metric, MI to avoid restricting the type of relationships within the sub-population. But another significant extension that differentiates NetExtractor to the other two techniques is that we calculate the inter-cluster score between the sub-populations that mandates the differential expression within samples. This separates NetExtractor outputs significantly from the previous algorithms since the GCN now constructed focuses on a network that have non-linearly related differentially expressed samples. The utility and novelty of NetExtractor lies in the fact that it is capable of detecting edges that would be conventionally missed by WGCNA and KINC. We do not claim that NetExtractor is ‘better’ than either of those methods but rather acts as an integral complement that extends the gene relationship network with edges that have biological relevance but have not been explored before.

As the results of our comparison between the three techniques we observe that although there is a significant overlap in the genes that are detected, it can be observed that the edges that are included in the GCN for each of the algorithm show variation. This outcome is expected since each of the algorithm inherently looks for a different kind of a relationship within the edges. These results indicate that neither of the algorithms is necessarily better or superior to the other but are trying to detect fundamentally different forms of relationships. We also performed functional enrichment analysis on the genes obtained in WGCNA_GCN and KINC_GCN in a manner similar to the one described above and found the enriched GO terms to be ’brain specific’. The enrichment results are included in Supplemental Table S4.

## Discussion

The implementation of GMMs to evaluate the expression distribution of different tissue samples between two genes permitted us to address the problem of extrinsic noise as well as determine if there are sub-populations of sample distribution within the edge that are interesting. Combining GMMs with MI further allowed us to explore non-linear relationships that may be embedded within the distribution. The NetExtractor workflow focuses on filtering out all edges that do not contain GMM sub-populations since in this work we focused on differentially expressed high MI binary RNA profiles. The remaining edges are further restricted by the inter-cluster loss criteria and then categorized into 5 clusters depicted in Figure 4. As noted earlier, clusters containing edges with either 1 or both genes exhibiting lower expression values for cerebellar *vs.* other brain tissues when both genes had differentially expressed samples did not show significant functional enrichment, where as clusters 1, 2, and 4 seemed to be significantly enriched for ’brain specific’ terms. This indicates that gene pairs that exhibit higher expression values for cerebellar tissues than other brain tissues are indicative of genes that are either more brain related or more specifically cerebellar tissue related.

Using these results as well as the extracted cerebellar high genes, it was then possible for us to utilize the PFC GRN PsychENCODE information in a novel methodology to construct a cerebellar specific GRN, which we validated using functional enrichment as well as by comparing our gene set for active histone acetylation peaks. The functional enrichment for the final 636 cerebellar GRN genes gave significant enriched terms in all categories with significance threshold of q value < 0.0001: *GO-MF* = 50, *GO-CC* = 27, *GO-BP* = 153, and *Human Phenotype* = 100. The top 5 terms and their respective IDs for various categories are depicted in Table 3 to illustrate the types of enrichment terms that were observed.

The active histone acetylation peaks experiments for the cerebellar GRN genes when compared against random or PFC high genes from the PsychENCODE GRN depict clear trends for the NetExtractor detected genes to be associated with actively transcribed genomic regions in cerebellar or cerebellar hemisphere tissues. This evidence is further bolstered when the cerebellar genes were compared against a random gene set for non-brain cell lines that showed a mostly consistent decrease in active histone acetylation peaks. Hence, utilizing the NetExtractor algorithm we were not only able to identify edges that were more likely to be associated to cerebellar tissues, using a novel method of separating PFC and cerebellar genes we were able to construct a GRN for cerebellar associated genes involving TFs connected to target genes. This cerebellar GRN can be utilized to identify unique cerebellar pathways as well as links that have not been explored completely.

Future directions for this project are twofold. Instead of restricting our analysis to only genes that behaved both as target genes as well as TF and constructing the GRN using those central nodes, we will focus on all the identified 808 cerebellar high genes and build a larger cerebellar GRN that will permit us to study and explore regulatory pathways in more depth. The second thrust for future works involves evaluating the GRN as well as the larger GRN in context with published protein-protein interaction (PPI) networks as well as superimposing networks of known microRNAs to better understand the regulatory pathways. On a broader scope, we envision the application of the NetExtractor workflow on larger GEMs such as panGTEx and panTCGA that may contain interesting non-linear sample expression distribution patterns for gene pairs.

### Limitations

Although NetExtractor demonstrated the capability of identifying edges that display non-linear patterns in distributed sub-populations of samples for the brainGTEX GEM, we recognize that it may not be a suitable fit for data that is not differentially expressed for a specific tissue type. This may lead to aberrant results that identify edges but may not be truly meaningful. Even so, from 1,579,304,301 edges NetExtractor was able to extract 10,966 edges, and then from that a meaningful set of 808 genes that seemed to be closely associated to a specific tissue type. It is especially important to note that NetExtractor is knowledge independent, *i.e.*, the algorithm itself is not cognizant of tissue annotations per sample.

Another drawback is that since we begin with a GRN from PsychENCODE created specifically for PFC tissues, we are limited to the genes and transcription factors that are contained within that network and cannot construct a fully robust cerebellar GRN. However, we demonstrate a novel technique to extract a sub-network relevant to the tissue of interest from the available GRN.

## Conclusion

In this report we detail an algorithm, NetExtractor, that combines the GMM along with MI to address limitations of current GCN construction techniques. The application of GMMs enabled the discovery of embedded sub-populations with the expression levels of the tissue samples while addressing the intrinsic and extrinsic noise obfuscating the signal. Furthermore, using MI to determine the dependency between the bigenic expression profile led to the isolation of non-linear relationships within an edge. We applied NetExtractor to a human brain GTEx brain GEM to construct a gene relationship network associated with cerebellar and cerebellar hemisphere tissue. Coupling these cerebellar edges with the PsychENCODE PFC GRN, we built a unique cerebellar GRN that should have utility in the study of gene regulation in the human cerebellum.

## References

[bib1] Barman S., and Kwon Y.-K., 2017 A novel mutual information-based boolean network inference method from time-series gene expression data. PLoS One 12: e0171097 10.1371/journal.pone.0171097 28178334 PMC5298315

[bib2] Bernstein B. E., Kamal M., Lindblad-Toh K., Bekiranov S., Bailey D. K., 2005 Genomic maps and comparative analysis of histone modifications in human and mouse. Cell 120: 169–181. 10.1016/j.cell.2005.01.001 15680324

[bib3] Buitinck L., Louppe G., Blondel M., Pedregosa F., Mueller A., 2013 API design for machine learning software: experiences from the scikit-learn project. In *ECML PKDD Workshop: Languages for Data Mining and Machine Learning*, pp. 108–122.

[bib4] Butte A. J., Tamayo P., Slonim D., Golub T. R., and Kohane I. S., 2000 Discovering functional relationships between rna expression and chemotherapeutic susceptibility using relevance networks. Proc. Natl. Acad. Sci. USA 97: 12182–12186. 10.1073/pnas.220392197 11027309 PMC17315

[bib5] Chan T. E., Stumpf M. P., and Babtie A. C., 2017 Gene regulatory network inference from single-cell data using multivariate information measures. Cell Syst. 5: 251–267.e3. 10.1016/j.cels.2017.08.014 28957658 PMC5624513

[bib6] Chen J., Bardes E. E., Aronow B. J., and Jegga A. G., 2009 Toppgene suite for gene list enrichment analysis and candidate gene prioritization. Nucleic Acids Res. 37: W305–W311. 10.1093/nar/gkp427 19465376 PMC2703978

[bib7] Davies E., 1978 Information and quantum measurement. IEEE Trans. Inf. Theory 24: 596–599. 10.1109/TIT.1978.1055941

[bib8] Eisen M. B., Spellman P. T., Brown P. O., and Botstein D., 1998 Cluster analysis and display of genome-wide expression patterns. Proc. Natl. Acad. Sci. USA 95: 14863–14868. 10.1073/pnas.95.25.14863 9843981 PMC24541

[bib9] Faith J. J., Hayete B., Thaden J. T., Mogno I., Wierzbowski J., 2007 Large-scale mapping and validation of escherichia coli transcriptional regulation from a compendium of expression profiles. PLoS Biol. 5: e8 10.1371/journal.pbio.0050008 17214507 PMC1764438

[bib10] Ficklin S. P., Dunwoodie L. J., Poehlman W. L., Watson C., Roche K. E., 2017 Discovering condition-specific gene co-expression patterns using gaussian mixture models: A cancer case study. Sci. Rep. 7: 8617 10.1038/s41598-017-09094-4 28819158 PMC5561081

[bib11] Gibson S. M., Ficklin S. P., Isaacson S., Luo F., Feltus F. A., 2013 Massive-scale gene co-expression network construction and robustness testing using random matrix theory. PLoS One 8: e55871 10.1371/journal.pone.0055871 23409071 PMC3567026

[bib12] Greenfest-Allen E., Cartailler J.-P., Magnuson M. A., and Stoeckert C. J., 2017 iterativewgcna: iterative refinement to improve module detection from wgcna co-expression networks. bioRxiv (Preprint Posted December 14, 2017). 10.1101/234062

[bib13] Hoadley K. A., Yau C., Hinoue T., Wolf D. M., Lazar A. J., 2018 Cell-of-origin patterns dominate the molecular classification of 10,000 tumors from 33 types of cancer. Cell 173: 291–304.E6. 10.1016/j.cell.2018.03.022 29625048 PMC5957518

[bib14] Husain B., and Feltus F. A., 2019 Edgescaping: Mapping the spatial distribution of pairwise gene expression intensities. PLoS One 14: e0220279 10.1371/journal.pone.0220279 31386677 PMC6684082

[bib15] Langfelder P., and Horvath S., 2008 Wgcna: an r package for weighted correlation network analysis. BMC Bioinformatics 9: 559 10.1186/1471-2105-9-559 19114008 PMC2631488

[bib16] Liang M., Zhang F., Jin G., and Zhu J., 2015 Fastgcn: a gpu accelerated tool for fast gene co-expression networks. PLoS One 10: e0116776 10.1371/journal.pone.0116776 25602758 PMC4300192

[bib17] Lonsdale J., Thomas J., Salvatore M., Phillips R., Lo E., 2013 The genotype-tissue expression (gtex) project. Nat. Genet. 45: 580–585. 10.1038/ng.2653 23715323 PMC4010069

[bib18] Lopes R. H., Reid I., and Hobson P. R., 2007 The two-dimensional kolmogorov-smirnov test. https://pdfs.semanticscholar.org/1cf6/fa61f4d7c2fc2848822a274ed07ee69889a59.pdf

[bib19] Peng H., Long F., and Ding C., 2005 Feature selection based on mutual information criteria of max-dependency, max-relevance, and min-redundancy. IEEE Trans. Pattern Anal. Mach. Intell. 27: 1226–1238. 10.1109/TPAMI.2005.159 16119262

[bib20] Pepke S., and Ver Steeg G., 2017 Comprehensive discovery of subsample gene expression components by information explanation: therapeutic implications in cancer. BMC Med. Genomics 10: 12 10.1186/s12920-017-0245-6 28292312 PMC5351169

[bib21] Petereit J., Smith S., Harris F. C., and Schlauch K. A., 2016 petal: Co-expression network modelling in r. BMC Syst. Biol. 10: 51 10.1186/s12918-016-0298-8 27490697 PMC4977474

[bib22] Poehlman W. L., Rynge M., Balamurugan D., Mills N., and Feltus F. A., 2017 Osg-kinc: High-throughput gene co-expression network construction using the open science grid. In *2017 IEEE International Conference on Bioinformatics and Biomedicine (BIBM)*, pp. 1827–1831. 10.1109/BIBM.2017.8217938

[bib23] Rapaport F., Khanin R., Liang Y., Pirun M., Krek A., 2013 Comprehensive evaluation of differential gene expression analysis methods for rna-seq data. Genome Biol. 14: 3158 10.1186/gb-2013-14-9-r95 PMC4054597 24020486

[bib24] Roche K., Feltus F. A., Park J. P., Coissieux M.-M., Chang C., 2017 Cancer cell redirection biomarker discovery using a mutual information approach. PLoS One 12: e0179265 10.1371/journal.pone.0179265 28594912 PMC5464651

[bib25] Rousseeuw P. J., 1987 Silhouettes: a graphical aid to the interpretation and validation of cluster analysis. J. Comput. Appl. Math. 20: 53–65. 10.1016/0377-0427(87)90125-7

[bib26] Roy A., Pordes R., and Altunay M., 2007 The open science grid.

[bib27] Wang D., Liu S., Warrell J., Won H., Shi X., , 2018 Comprehensive functional genomic resource and integrative model for the human brain. Science 362: eaat8464. 10.1126/science.aat8464 PMC6413328 30545857

[bib28] Wang J., Chen B., Wang Y., Wang N., Garbey M., 2013 Reconstructing regulatory networks from the dynamic plasticity of gene expression by mutual information. Nucleic Acids Res. 41: e97 10.1093/nar/gkt147 23470995 PMC3632132

[bib29] Zerbino D. R., Achuthan P., Akanni W., Amode M. R., Barrell D., 2017 Ensembl 2018. Nucleic Acids Res. 46: D754–D761. 10.1093/nar/gkx1098 PMC5753206 29155950

[bib30] Zhang X., Zhao J., Hao J.-K., Zhao X.-M., and Chen L., 2014 Conditional mutual inclusive information enables accurate quantification of associations in gene regulatory networks. Nucleic Acids Res. 43: e31 10.1093/nar/gku1315 25539927 PMC4357691

